# Dr. Oscar J. Coskery

**Published:** 1889-08

**Authors:** 


DR. OSCAR J. COSKERY, the accomplished professor of surgery in the Baltimore College of Physicians and Surgeons, died of phthisis in the city of his home on the 5th of July, 1889, aged 46 years.

---

THE Brown-Sequard system of rejuvenation, though the hypodermic injection of testicular juices, is attracting the attention of the non-professional, or so-called secular press. Well, most of the overworked and worn-out editors need mental and physical rehabilitation, and we hope, in this new-found fountain of life, they will bathe and be restored.

